# Combination of Hairy Root and Whole-Plant Transformation Protocols to Achieve Efficient CRISPR/Cas9 Genome Editing in Soybean

**DOI:** 10.3390/plants12051017

**Published:** 2023-02-23

**Authors:** Qihui Kong, Jie Li, Shoudong Wang, Xianzhong Feng, Huixia Shou

**Affiliations:** 1 State Key Laboratory of Plant Physiology and Biochemistry, College of Life Sciences, Zhejiang University, Hangzhou 310058, China; 2 Zhejiang Lab, Hangzhou 310012, China; 3 Key Laboratory of Soybean Molecular Design Breeding, Northeast Institute of Geography and Agroecology, Chinese Academy of Sciences, Changchun 130102, China

**Keywords:** soybean transformation, CRISPR/Cas9, hairy root, genome-editing

## Abstract

The new gene-editing technology CRISPR/Cas system has been widely used for genome engineering in various organisms. Since the CRISPR/Cas gene-editing system has a certain possibility of low efficiency and the whole plant transformation of soybean is time-consuming and laborious, it is important to evaluate the editing efficiency of designed CRISPR constructs before the stable whole plant transformation process starts. Here, we provide a modified protocol for generating transgenic hairy soybean roots to assess the efficiency of guide RNA (gRNA) sequences of the CRISPR/Cas constructs within 14 days. The cost- and space-effective protocol was first tested in transgenic soybean harboring the GUS reporter gene for the efficiency of different gRNA sequences. Targeted DNA mutations were detected in 71.43–97.62% of the transgenic hairy roots analyzed as evident by GUS staining and DNA sequencing of the target region. Among the four designed gene-editing sites, the highest editing efficiency occurred at the 3′ terminal of the GUS gene. In addition to the reporter gene, the protocol was tested for the gene-editing of 26 soybean genes. Among the gRNAs selected for stable transformation, the editing efficiency of hairy root transformation and stable transformation ranged from 5% to 88.8% and 2.7% to 80%, respectively. The editing efficiencies of stable transformation were positively correlated with those of hairy root transformation with a Pearson correlation coefficient (r) of 0.83. Our results demonstrated that soybean hairy root transformation could rapidly assess the efficiency of designed gRNA sequences on genome editing. This method can not only be directly applied to the functional study of root-specific genes, but more importantly, it can be applied to the pre-screening of gRNA in CRISPR/Cas gene editing.

## 1. Introduction

Soybean [*Glycine max* (L.) Merr.] is an economically important crop for feed, oil, and protein products. It contains about 40% protein and 20% oil in the seed [1,2]. In the past decades, significant progress has been made in soybean functional genomics and its application in molecular breeding [3,4,5]. Genome editing is a tremendous strategy for efficient and targeted genome manipulations, especially for crops that have complex genomes and difficulty being improved through conventional breeding approaches [6,7]. The targeted genome editing methods have undergone three generations of technological development and improvement, from zinc-finger nucleases (ZFNs) to transcription activator-like effector nucleases (TALENs), and recently the clustered regularly interspaced short palindromic repeats (CRISPR)/CRISPR-associated protein (CRISPR/Cas) [8]. All three methods induce double-stranded breaks in the target genome DNA, which are subsequently repaired through non-homologous end joining and homologous recombination [9]. While ZFNs and TALENs target the genome through protein–DNA interactions, the CRISPR/Cas system is based on short RNA–DNA base pairing [10,11,12]. Compared to TALENs and ZFNs, the CRISPR/Cas toolkit is simple to design as it involves only single-stranded guided RNA (gRNA) and the Cas protein. CRISPR-Cas genome editing technology has attracted great attention and has been successfully applied in various crops for functional genomic study and molecular breeding [13]. 

CRISPR/Cas system has been successfully adapted in various plant species, including soybean [14,15,16,17,18,19,20,21]. The system requires the efficient delivery of the CRIPSR construct into germline cells and regeneration of the edited germline cells into plants. In soybean, the whole-plant transformation often uses *Agrobacterium-tumefaciens*-meditated cotyledonary node protocol [22,23]. Although the protocol has been improved in the past decades, it is still time-consuming, has a low efficiency, and is highly dependent on skilled operators. Furthermore, approximately 75% of soybean genes are present in multiple copies [24], which increases the complexity of efficient gene editing. A practical system to assess the effectiveness of the designed gRNAs on gene editing prior to apply the gRNAs into whole-plant transformation is of importance.

Compared to *A. tumefaciens*-mediated whole-plant transformation, *A. rhizogenes*-based root hairy transformation is a simple and efficient transformation system to achieve transgenic roots. *A. rhizogenes* can transfer the “Transferred DNA” segment from its root-inducing plasmid into the genome of the host plant [25,26]. The transfer DNA consists of *rol* gene to induce the formation of root-like structures known as hairy roots. Hairy root-based CRISPR/Cas9 genome editing was successfully used to study the gene function, especially in the species that are recalcitrant to transformation [27]. Jacob et al. tested the editing efficiencies of CRISPR/Cas9 vectors to knock out the *GFP* gene in GFP-expressing soybean line via hairy root transformation [28]. By observing the loss of fluorescence, they found that 15 out of 17 5′-target events and 4 of the 22 3′-target events were knockouts [28]. The results also indicated that the CRISPR system is able to modify both GFP alleles so that completely diminished the fluorescence in the GFP homozygous line. The study also tested the knockout efficiencies on the single-copy soybean gene *Glyma07g14530* and identified a variety of mutations, including deletions, SNPs, insertions, and replacements [28]. Another attempt used *A. rhizogenes* as a tool for fast and efficient visualization of the expression pattern of the transcription factors *SHORTROOT* (*SHR*) and *SCARECROW* (*SCR*) gene in roots of *S. lycopersicum* and *S. pennellii* [29]. In addition, the hairy root transformation was also used in the characterization of promoters, functional analysis of root-specific genes, and the production of valuable phytochemicals [27,30,31,32]. 

CRISPR/Cas9-mediated genome editing is easier to implement than other loss-of-function tools in polyploid plant species, where most genes have homeologs. CRISPR/Cas9 editing tools could be used to target a single or multiple homoeologous genes at the same time [27,28]. The homoeologous gene pair *01gDDM1* and *011gDDM1* was targeted singly or at the same time using the hairy root transformation method [28]. The vector targeting both homoeologous gene pairs resulted in low average editing frequencies, with 21% and 8.9% for the chr1 and chr11 targets, respectively. However, the editing efficiency for a different homoeologous gene pair was greater than 97%, suggesting that the lower indel frequency of the 01g + 11g DDM1 vector is due to the gRNA itself and not a result of targeting multiple genes at once [27]. 

In summary, although the hairy root transformation system has been widely used in soybean gene editing, the comparison of its editing efficiency with that of whole plant transgenes and the application of this system in the evaluation of candidate gRNA efficiency have not been reported. In this study, by using the imbibed seeds as explants, we were able to reduce the duration of hairy root transformation procedure from the classic protocol of 3–4 weeks to 2 weeks. Thus, the protocol is used to rapidly evaluate the effectiveness of the CRISPR/Cas9 constructs. Our data showed that the editing efficiencies of different gRNAs in hairy root and whole-plant transformation ranged from 5.0% to 88.8%, and 2.7% to 80.0%, respectively. The editing efficiency of stable transformation was positively correlated with those of hairy root transformation with a Pearson correlation of efficient (r) of 0.83. Meanwhile, we efficiently edited the targeted GUS and soybean endogenous *PDS* genes for editing in hairy roots. Using the pre-screening of gRNAs, we were able to achieve whole-plant editing events for 26 soybean genes, which provides a potential way to study soybean gene function and genetic improvement.

## 2. Results

### 2.1. A Rapid and Efficient Protocol for A. rhizogenes-Mediated Hairy Root Transformation System in Soybean

**Figure 1:**
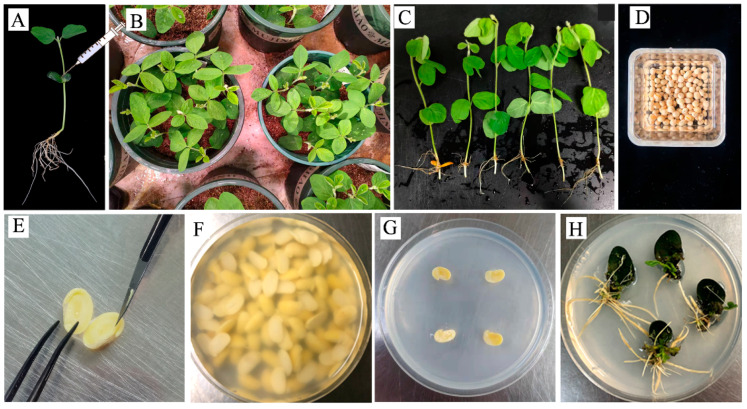
Procedures of soybean hairy root transformation using one-week-old seedlings or imbibed seeds as explants. (**A**–**C**) Procedure of *A. rhizogenes*-mediated hairy root transformation using one-week-old seedlings as explants. (**A**) Soybean seeds were plants in vermiculite soil pots for one week prior to inoculation. (**B**) Inoculation of *A. rhizogenes* by stabbing of hypocotyls adjacent to cotyledonary nodes. (**C**) Induction of hairy roots 2 weeks post-inoculation (DPI). (**D**–**H**) Procedure of *A. rhizogenes*-mediated hairy root transformation using imbibed seedlings as explants and adding a select agent in root induction medium (**D**) Overnight imbibed seeds as explants. (**E**) Preparation of half-seed explants. (**F**) Inoculation of *A. rhizogenes*. (**G**) Explants in the root inducing (RI) medium. (**H**) Induced hairy roots at 14 days post-infection.

To use the hairy root transformation system to assess of CRISPR/Cas9 constructs, we first made efforts to optimize the hairy root transformation protocol for its robustness and effectiveness. Previously, hairy root transformation was performed by stabbing the hypocotyl of soybean seedlings as described [33]. By inoculating hypocotyls adjacent to cotyledonary nodes of the one-week-old seedlings with *A. rhizogenes* stain K599, transgenic hairy roots were obtained in 4 weeks (Figure 1A–C). 

To improve the robustness and effectiveness of the hairy root transformation protocol, we tried split-imbibed soybean seeds as explants for the *A. rhizogenes*-mediated hairy root transformation. The explant preparation method was referred from the soybean cotyledenary nodes whole-plant transformation [22,23]. In brief, the overnight imbibed seeds were excised into two half-seeds, then inoculated with *A. rhizogenes* suspension culture for 30 min. The infected cotyledons were then transferred to root induction (RI) medium to produce hairy roots. Hairy roots emerged at 6 days post-inoculation (DPI). By 14 DPI, the transgenic roots had grown to 1–2 cm, which is suitable for DNA extraction and further identification (Figure 1D–H). 

**Figure 2:**
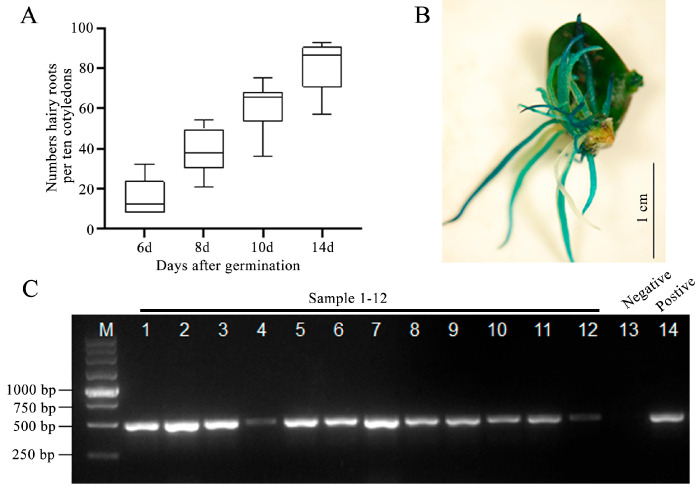
Generation and verification of transgenic hairy roots. (**A**) Numbers of hairy roots generated at different days post-*A. rhizogenes* inoculation. Hairy root numbers of each ten cotyledon were calculated. (**B**) GUS-staining assay of hairy roots at 14 days after germination. Bars = 1 cm. (**C**) PCR identification of positive hairy roots. Lane 1–12, GUS-positive roots; lane 13, negative control; lane 14, positive control, respectively.

To verify the effectiveness of the protocol, the binary plasmid pTF102 [23] was introduced into *A. rhizogenes* strain K599 to overexpress GUS reporter gene in hairy roots. The number of generated hairy roots per explant was calculated at 6, 8, 10, and 14 DPI. Putative transgenic roots started to emerge at 6 DPI and increased to an average of 8.4 at 14 DPI (Figure 2A). GUS (*β*-glucuronidase) staining and polymerase chain reaction (PCR) verification of each hairy root generated from explants were carried out. Results showed that nearly all regenerated hairy roots are PCR- and GUS-positive (Figure 2B,C). The transformation frequency reached 94.3% with the RI medium containing 5 mg/L glufosinate as the selecting agent. 

In summary, by using the imbibed seeds as explants, we were able to reduce the duration of the hairy root transformation procedure from the classic protocol of 3–4 weeks to 2 weeks. Since Ri medium contains the selective agent, the vast majority of the hairy roots obtained are transgenic.

### 2.2. CRISPR/Cas9-Induced Mutagenesis of GUS Transgene in Soybean Hairy Roots

**Figure 3:**
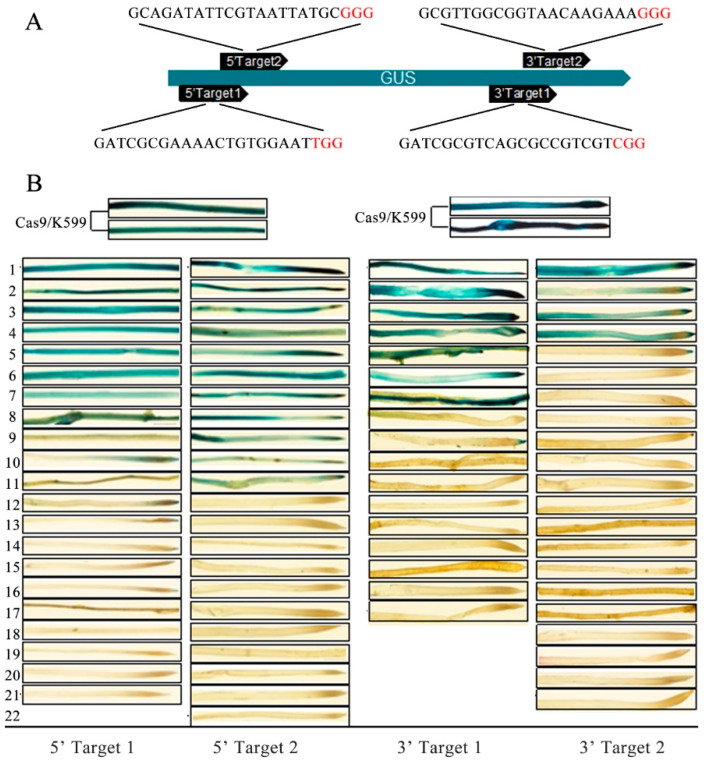
The generating of CRISPR-edited transgenic roots in GUS-containing transgenic soybean. (**A**) Location and target sequences of gRNAs in the GUS gene. Two gRNAs were designed to target the 5′ end of GUS (5′-target), and the other two were designed to target the 3′ end (3′-target). Black arrows are GN20GG target motifs. PAM (NGG) sequences are highlighted in red. Target site length was 20 nt. (**B**) GUS staining of the hairy roots generated from CRISPR-edited transgenic roots. Cas9/K599 is *A. rhizogenes* stain containing a construct with Cas9 expression cassette and without a gRNA.

Although whole-plant transformation of soybean has become routine, the transformation efficiency is often low and the procedure to obtain transformed plantlets takes at least three months. Thus, using hairy root system to assess the effective target gRNA prior to conducting the whole-plant genome editing would be greatly save time and costs. To evaluate the gene-editing efficiency of different target designs of the GUS reporter gene, four GUS-CAS9 constructs with different gRNA sequences were made to edit the GUS gene in the GUS-expressing soybean lines [23]. The GUS-expressing soybean lines are the homozygous transgenic soybean containing a single copy of GUS expression cassette driven by the CaMV35S promoter. Four gRNAs were designed to target the 5′ end of GUS (5′Target1 and 5′Target2) or the 3′ end (3′Target1 and 3′Target2) (Figure 3A). The constructs were introduced into *A. rhizogenes* stain K599 to produce transformed hairy roots, in which the previously transferred GUS-coding sequence was edited. The success of *GUS* editing was evident by both GUS staining and sequencing of target region. While the transgenic roots generated from the control construct containing only the Cas9 expression cassette without gRNA were stained dark blue (the top rows in Figure 3B), GUS staining of the transgenic roots showed significant reduction in blue color (Figure 3B). When both GUS alleles were edited, no blue color was seen in GUS staining. 

The GUS gene-editing efficiency was calculated by the number of edited GUS alleles out of the total GUS copies. The generated transgenic hairy roots include three types, i.e., not edited (0 of 2 alleles edited), heterozygous mutant (1 of 2 alleles edited) and homozygous mutant (2 alleles edited). The editing rates were 71.43%, 77.27%, 79.41%, and 97.62% for the gRNAs of 5′Target1, 5′Target2, 3′Target1 and 3′Target2, respectively (Figure 3B, Table 1).

To verify the knockouts and determine the genetic modifications of the transgenic roots, Sanger sequencing was carried out on the amplicons that amplified using the PCR primer pairs covering the target regions. Results showed that the most common mutation were short (1–32 nt) (Figure S1). In addition, sequence insertion and (up to 35 bp), and base substitution were also commonly seen (Figure S1).

### 2.3. Gene-Editing Efficiency of the Two Homeologous Genes of Soybean Phytoene Dehydrogenase (PDS)

**Figure 4:**
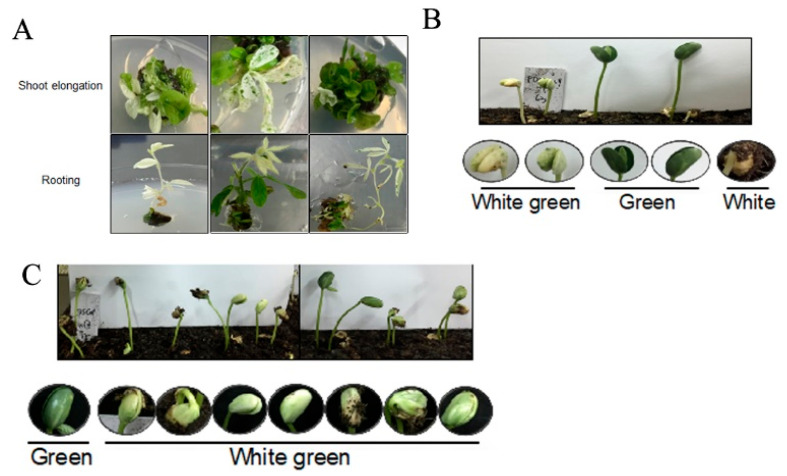
Segregation of the *GmPDS* mutation. (**A**) Phenotype of CRISPR/Cas9-mediated *GmPDS* knockout mutant during shoot elongation and rooting. (**B**) Segregation of the T_1_ seeds harvested from T_0_ *GmPDS* heterogenous mutant. (**C**) Segregation of the T_2_ seeds harvested from T_1_ *GmPDS* heterogenous mutant.

Soybean has a paleopolyploid genome, in which nearly 75% of genes are present in multiple copies [24]. To evaluate the efficiency of the CRISPR/Cas9 system in editing the duplicated genes simultaneously, we first designed gRNAs targeting the common sequence of the two soybean phytoene dehydrogenase (*GmPDS*) genes, *GmPDS1* (*Glyma.11G253000*) and *GmPDS2* (*Glyma.18G003900*). Phytoene desaturase plays critical functions in pigment synthesis, and disruption of its functions leads to albino plants [34]. Five gRNAs were designed and evaluated in hairy root transformation system with various gene-editing efficiencies ranging from 35.6 to 100.0%. Then, we selected *GmPDS1/2 gRNA-1, GCATTAATGATCGGTTACAATGG*, which showed 100% *PDS* gene-editing efficiency in hairy root, to construct CRISPR/Cas9 vector for stable soybean transformation. Twenty-six Cas9-positive T_0_ plants were generated. Sequencing of the target region of *GmPDS* genes showed that among 26 Cas9-positive events, 7 events had mutation on *GmPDS2*, and 4 events had mutation in both *GmPDS1* and *GmPDS2*, with the gene-editing efficiencies for *GmPDS2* only and both genes of 26.9% and 15.4%, respectively. The *pds1/2* mutants with both *GmPDS1* and *GmPDS2* gene edited showed bleached leaf color, which can be observed during shoot elongation, rooting, and the seedling stages (Figure 4A). Sequencing of the four events with both *GmPDS* alleles edited showed that the editing patterns included base deletion from −1 to −11, base addition from +1 to +23, and a base substitution of 3 (Table 2, Figure S1). As the *GmPDS* homozygous mutant seedlings cannot survive, the white-green heterozygous mutants that contain at least one wild-type allele of *GmPDS1* or *GmPDS*2 were used for the segregation assay. Results show that the cotyledon color of the T_1_ seedlings of *pds* heterozygous mutant follows a Mendelian segregation pattern (Figure 4B,C).

### 2.4. Gene-Editing Efficiency of Different Targets of Soybean Transparent Testa 8a (GmTT8a) and GmTT8b 

The described hairy root transformation system was applied and evaluated in editing the pair of soybean function genes, *GmTT8a* (*Glyma.02G147800*) and *GmTT8b* (*Glyma.10G026000*). In this attempt, gRNAs were designed for targeting to the common region of both *GmTT8a* and *GmTT8b*, or targeting to either of *GmTT8a* and *GmTT8b* alone. PCR primers were selected in the locus-specific region to distinguish the mutations in the different locus.

A total of 10 gRNAs were designed, including 2 for both of *GmTT8a* and *GmTT8b*, 3 for *GmTT8a*, and 5 of *GmTT8b* (Table 3). The mutation rate of these targets was calculated as the number of hairy roots containing edited sequence at the target site divided by the total number of hairy roots that contain the selectable marker gene *bar* and *Cas9* gene (Table 3). Editing of the target region was achieved in 6 out of the 10 gRNAs, including *GmTT8a/b-1, GmTT8a-2, -3,* and *GmTT8b-2, -3, -5,* with different editing efficiency ranging from 15% to 100% (Table 3). Based on the data, we selected *GmTT8a/b-1, GmTT8a-2, and GmTT8b-2* as the gRNAs for the whole-plant transformation. As showed in Table 3, using *GmTT8a-2 and GmTT8b-2* gRNAs, we were able to obtain five and eight whole plant mutants for *GmTT8a* and *GmTT8b*, respectively. No double mutant of *GmTT8a* and *GmTT8b* were identified among the 30 Cas9 positive transgenic events. It is possible that knocking out both *GmTT8a* and *GmTT8b* caused lethality.

### 2.5. Achievement of Efficient CRISPR/Cas9-Induced Targeted Mutations of Other Functional Genes in Soybean

We applied the hairy root transformation system to pre-screening the efficient gRNA for the whole-plant genetic transformation. We collected different experiments in the lab to prove the importance of pre-screening of targets using hairy root system. Table 4 showed the overall CRISPR/Cas9 gene-editing events for 13 gRNAs involving six different pairs of soybean homoeologous genes. Experimental data of the gRNA1, 2, and 4 are collected from published studies from the lab [35,36,37]. The gRNAs 5-1 and 6-3 were failed to achieve any transgenic hairy roots so that they were abandoned for stable transformation (Table 4). The target regions of the transgenic hairy roots obtained from the gRNAs 5-2 and 6-2 were detailed listed (Figure S3). Among the gRNAs selected for stable transformation, the editing efficiencies of hairy root transformation and stable transformation ranged from 5.0 to 88.8% and 2.7% to 60.0%, respectively (Table 4). The editing efficiencies of stable transformation were positively correlated with those of hairy root transformation with a coefficient of determination (R2) of 0.70, i.e., a Pearson correlation coefficient of 0.83 (Supplementary Table S1).

By pre-screening the effective gRNAs, we successfully obtained gene-editing mutants from 16 gRNA, which targeted 10 pairs of soybean homeologs and four single-copy genes (Table 4 and Table 5). The gene-editing efficiencies ranged from 2.7% of gRNA6-1 (Table 4) to 80.0% of gRNA9-1 (Table 5). 

We calculated the efficiency of simultaneous editing of two homeologs by a single gRNA in the CRISPR/Cas9 system. A total of 13 gRNAs (gRNA1-1, 2-1, 3-1, 4-1, 4-2, 5-1, 5-2, 6-1, 6-2, 7-1, 8-1, 9-1, and 10-1, Table 4 and Table 5), each targeting a pair of soybean homeologs simultaneously, were tested for their editing efficiencies in the whole-plant transformation system. Twelve of the thirteen gRNAs produced corresponding double mutants with sequence editing in both alleles (Table 4 and Table 5). Therefore, editing a pair of homeologs in the soybean genome with a single gRNA can be very efficient, unless simultaneous editing of a pair of genes causes lethal problem.

## 3. Discussion

In the study, by using the imbibed seeds as explants, the duration of soybean hairy root transformation procedure was reduced to 2 weeks (Figure 1), which is at least 1 week shorter than the classic seedling-stabbing protocol [33]. A significant positive correlation was detected between the editing efficiencies of gRNAs in hairy root transformation and whole-plant transformation (Table S1). Thus, the modified hairy root transformation protocol can be used to assess the editing efficiency of designed gRNAs prior to whole-plant transformation in soybean. 

### 3.1. The Modified Hairy Root Transformation System Not only Shortens the Duration, but also Guarantees the Ratio of Transgenic Roots in the Obtained Hairy Roots

In this study, we used split-seeds as explants for hairy root transformation. The explant preparation method was referred from the *A. tumefaciens*-mediated whole soybean plant transformation procedure [22,23]. Germline cells infected and transformed by *A. rhizogenes* here are the same as the reported whole-plant transformation protocol, that is, the cotyledons axillary bud primordium. The major differences between this modified protocol and the classic seedling stabbing protocol [33] include the following: (1) the explants used in our protocol have undergone only 16 h of immersion; (2) the selective agent corresponding to the resistance marker gene contained in the binary vector was added to the Ri medium. As a result, the modification not only shortens the duration of obtaining transgenic roots by one week, but also ensures that the hairy roots obtained are produced by T-DNA insertion. Using the modified protocol, the verification of the hairy roots obtained can then be eliminated. 

### 3.2. Impartance of Pre-Evaluation of the Editing Efficiency of gRNAs in Hairy Root Transformation

The target sites for CRISPR/Cas9-based genome editing can be designed manually or assisted by website tools such as CRISPR-P [38] and CRISPR-GE [39]. However, the CRISPR/Cas9 system is unable to edit all targetable genomic sites with full efficiency in vivo due to the genome complexity. Thus, it is necessary to pre-screen the valid gRNAs before whole-plant transformation begins. In this study, 26 soybean genes were simultaneously edited in CRISPR/Cas9 based on hairy root and whole-plant transformation. This study, for the first time, compared the gene-editing efficiencies of a set of gRNAs in the two *Agrobacterium* species-mediated transformation systems. Result showed that the editing efficiency of a certain gRNA in whole-plant transformation is highly correlated with that in hairy root transformation with a Pearson correlation coefficient of 0.83 (Supplementary Table S1). The use of hairy root system to pre-evaluate the editing efficiency of gRNAs can also be applied to other plant species that are recalcitrant to plant transformation to avoid waste caused by poor gRNA design in the whole plant transgenic process. 

In this study, we proposed a new use of hairy root transformation system, which is to pre-assess the effectiveness of gRNAs designed in CRISPR/Cas9-mediated gene-editing vectors before the whole-plant transformation begins. The protocol can screen gRNAs for a better editing efficiency in the target within two weeks. The expected gene-editing plants could be successfully obtained through the transformation of whole soybean plants using the pre-selected gRNAs. 

Pre-assessment of gRNA editing efficiency can also be achieved more rapidly using simpler systems. For instance, using *Agrobacterium*-mediated tobacco infiltration [40], the gRNA and CAS9 proteins can be expressed transiently in tobacco leaves, and the editing efficiency of gRNAs in the targeting gene of the tobacco genome can be assessed within three days. A recent paper successfully used the engineered tomato-spotted wilt virus to deliver the CRISPR/Cas components in various plant species [41]. It provides a promising tool for gene editing in the plant species that are recalcitrant to tissue-culture-based plant transformation in the future.

## 4. Materials and Methods

### 4.1. Plant Materials and Growth Conditions

Soybean cultivar Williams 82 was used for gene-editing experiments. In the experiment, to test the editing efficiency of gRNAs targeting the transgene GUS, transgenic soybean (c.v. Williams 82) harboring a single copy of *35S::*GUS expression cassette was used. Soybean plants were grown in a greenhouse at 30 °C day/25 °C night and with cycles of 16 h of light/8 h of dark.

### 4.2. Vector Construction

The binary plasmid pTF102 [23] was used for over-expressing the GUS reporter gene. The vector contains the expression cassettes of a phosphinothricin acetyl transferase (*bar*) gene conferring resistance to herbicide phosphinothricin, an intron-containing GUS gene in its T-DNA region.

The CRISPR/CAS9 construct was based on the pBlu-gRNA vector and CAS9 MDC123 (Addgene plasmid # 59188 and 59184, Watertown, MA, USA). The target sites were designed using the webtool of http://skl.scau.edu.cn/ (accessed on 15 January 2023) [34]. The target sequences were synthesized and cloned into pBlu/gRNA at the *Bbs*I site and under the control of the U6 promoter. The construct was then digested with *Eco*RI to generate the gRNA cassette and inserted into destination vector CAS9 MDC123. The sequences of the analyzed soybean genes were downloaded from phytozome (https://phytozome-next.jgi.doe.gov/ (accessed on 15 January 2023)). The resulting constructs were named as “genename-CAS9”, e.g., *gus*-CAS9, *pds*-CAS9, *tt8a*-CAS9, and so on. To knockout the pair of the homoeologous genes, gRNA was designed in the common region of the genes. All the corresponding primers are listed in Supplementary Table S2.

### 4.3. Hairy Root Transformation

For hairy root transformation, constructs were transformed into *A. rhizogenes* strain K599 by the heat shock method. 

*A. rhizogenes*-mediated hairy root transformation was performed according to the literature [37]. The modified protocol uses imbibed seedlings as explants. Briefly, soybean seeds were surface-sterilized for 10–12 h using chlorine gas in a sealed desiccator. The sterilized seeds were imbibed in sterile water at 25 °C for 16 h (overnight). The cotyledons and hypocotyls (about 5 mm) from a single seed were cut evenly into two half-seeds. The prepared explants were then inoculated with *A. rhizogenes* strain of K599 containing the corresponding CRISPR/Cas9 gene-editing vectors for 30 min. After inoculation, the explants were evenly (adaxial side up) placed into root induction medium containing B5 salts and vitamins, 3% sucrose, 0.8% agar, 0.58 mg/L MES (pH 5.8), filter-sterilized 1.67 mg/L BAP, 250 mg/L cefotaxime, and 5 mg/L glufosinate (the selective agent for the vector), and incubated in a growth room at 25 °C. Hairy roots emerged at 6 DPI and grew to 1–2 cm long at 14 DPI. 

### 4.4. Whole Plant Transformation Experiment of Soybean

After assessing the editing efficiency in hairy roots, constructs containing the efficient gRNA were transformed into *A. tumefaciens* strain LBA4404. The stable transgenics soybean plants were generated via *A. tumefaciens* -mediated cotyledenary node protocol [23]. Soybean “Williams 82” was used as the transformation recipient. Transgenic plants were identified by selectable *cas9* gene amplification and leaf painting with glufosinate (135 mg/L).

### 4.5. Verification of CRISPR/Cas9-Induced Mutations in Transgenic Roots and Plants

To verify the knockouts and determine the genetic modifications in the transgenic roots or plants, Sanger sequencing was carried out on the amplicons that amplified using the PCR primer pairs covering the target regions. Briefly, genomic DNA of the transformed hairy roots or stable transgenic soybean plants was extracted using the TPS [100 mM Tris-HCl (pH 8.0), 10 mM EDTA (pH 8.0), and 1 M KCl] method. PCR amplification was performed using the primers listed in Supplementary Table S2. The reaction conditions were as follows: 95 °C for 2 min, 34× (95 °C for 10 s, 58 °C for 15 s, 72 °C for 15 s), 72 °C for 5 min.

Transgenic roots or plants were first verified for the existence of *Cas9* gene using the Cas9-F/R primers. Cas9 positive transformants were further amplified to obtain the 400~800 bp fragments covering the gRNA targeting region. PCR products were then purified and sequenced. When gRNA targets a common region of a pair of homologous genes, gene-specific PCR primers should be designed to distinguish the two genes.

### 4.6. Histochemical GUS Assays

The transgenic hairy roots were immersed in GUS-staining buffer (100 mM sodium phosphate, pH 7.0, 1 mM 5-bromo-4-chloro-3-indolyl-b-D-glucuronidase), 1 mM K_4_[Fe(CN)_6_], 1 mM K_3_[Fe(CN)_6_], 0.5% TritonX-100 and 20% methanol) at 37 °C for 12 h. Afterwards, samples were washed with 70% ethanol. Images were captured under a stereomicroscope (Eclipse 90i; Nikon, Tokyo, Japan).

## 5. Conclusions

In summary, we reported an efficient soybean hairy root transformation protocol. Using the modified protocol, transgenic hairy roots could be obtained within 2 weeks, which is at least 1 week shorter than the classic seedling-stabbing protocol. More importantly, we found that there is a significant positive correlation between the editing efficiency of gRNAs in hairy root transformation and whole-plant transformation. Thus, the modified hairy root transformation protocol can be used to assess the editing efficiency of designed gRNAs prior to whole-plant transformation in soybean.

## Figures and Tables

**Table 1:** Gene-editing efficiencies of different gRNAs targeting the GUS genes.

Targets	Sequence	Total	Gene Editing in Generated Hairy Roots			
			Not Edited	1 Allele Edited	2 Alleles Edited	Editing Rate (%)
5′ Target 1	GATCGCGAAAACTGTGGAATTGG	21	2	8	11	71.43%
5′ Target 2	GCAGATATTCGTAATTATGCGGG	22	2	6	14	77.27%
3′ Target 1	GATCGCGTCAGCGCCGTCGTCGG	17	2	3	12	79.41%
3′ Target 2	GCGTTGGCGGTAACAAGAAAGGG	21	0	1	20	97.62%

**Table 2:** Gene-editing patterns of the T_0_ plantlets with mutations in both *GmPDS1* and *GmPDS2*.

Events	Target Gene	Alleles	Target Sequence After Editing	Leaf Color of Seedlets
*pds-1*	*GmPDS1*	1	WT: GCATTAATGATCGGTTACAATGG	White green
		2	+1: GCATTAATGATCGGTTATCAATGG	
	*GmPDS2*	1	−3: GCATTAATGATCGGT- - - AATGG	
		2	−1: GCATTAATGATCGGTTA- AATGG	
*pds-2*	*GmPDS1*	1	WT: GCATTAATGATCGGTTACAATGG	White green
		2	−9: GCATTAATG- - - - - - - - -AATGG	
	*GmPDS2*	1	−2: GCATTAATGATCGGT- - CAATGG	
		2	−11: GCATTAAT- - - - - - - - - - - ATGG	
*pds-3*	*GmPDS1*	1	−13: GCAT- - - - - - - - - - - - - CAATGG	White green
		2	−9: GCATTAAT- - - - - - - - - CAATGG	
	*GmPDS2*	1	WT: GCATTAATGATCGGTTACAATGG	
		2	−2: GCATTAATGATCGGT- - CAATGG	
*pds-4*	*GmPDS1*	1	WT: GCATTAATGATCGGTTACAATGG	White green
		2	+23: GCATTAATGATCGGTTA TGCCAAATGGAAGATTTTTGCAACAATGG	
	*GmPDS2*	1	−1: GCATTAATGATCGGTTA-AATGG	
		2	3 subs: GCATTAATGATCGGTTAGCTTGG	

**Table 3:** Gene-editing efficiency of different gRNAs of *GmTT8a* and *GmTT8b*.

Target Gene	Targets	5′-3′ Sequence	Editing Efficiency in Hairy Root Transformation			Editing Efficiency in Whole-Plant Transformation		
			# Cas9+ Roots	# Roots Edited	Editing Rate (%)	# Cas9+ Plants	# Plants Edited	Editing Rate (%)
*GmTT8a/b*	1	GAAGACGGTGCAACAATGGAGG	20	3	15.0%	31	0	0.0%
	2	GTGTGCATTCCTTTATTGGACGG	20	0	0.0%	N/A		
*GmTT8a*	1	GAAGACGGTGCAACAATGGAGG	20	0	0.0%	N/A		
	2	GCTCACTGGTGCAAACGAGGTGG	17	14	82.4%	15	5	33.3%
	3	GCCCAGCGAGCTGATGCAGCTGG	10	6	60.0%	N/A		
*GmTT8b*	1	GAGGCACATCTATGGCTCACGG	15	0	0.0%	N/A		
	2	GCTCACGGGTCAAATGAGGTGG	12	12	100.0%	13	8	61.5%
	3	GTGTGCATTCCTTTATTGGACGG	10	8	80.0%	N/A		
	4	GGATATTGAGGAGGAAGAGAGG	12	0	0.0%	N/A		
	5	GAAGATGAGGAGCCGAATCTGG	9	7	77.8%	N/A		

Note: N/A, no available.

**Table 4:** Gene-editing efficiency of gRNA1-6 in hairy root and whole-plant transformation.

Ref.	gRNAs to Target Pair of Homeologs		Gene Locus	Hairy Root Transformation			Whole-Plant Transformation		
				# Cas9+ Roots	# Roots Edited	Editing Rate (%)	# Cas9+ Plants	# Plants Edited	Editing Rate (%)
[35]	gRNA1-1	GGCATAGTATAGCCAAAGCATGG	05G122200	21	15	71.4%	5	2	40.0%
			08G077200		12	57.1%		2	40.0%
[36]	gRNA2-1	GATCGAGTTGATCGTAATAAGGG	15G049200	23	14	60.8%	7	2	28.5%
			08G183500		16	69.5%		3	42.8%
Unpublished	gRNA3-1	GCATTTGCCTTCGGCATGCTAGG	18G301100	18	14	77.70%	5	3	60.0%
			08G360500		16	88.80%		2	40.0%
[37]	gRNA4-1	GGTGGTGGGCCTGCAAACCTTGG	05G012300	20	12	60.0%	14	3	21.4%
			17G012400		7	35.0%		1	7.1%
	gRNA4-2	GTTAAAAGTGCTGGGCTTCTTGG	05G012300	20	11	55.0%	28	4	14.3%
			17G012400		9	45.0%		3	10.7%
Unpublished	gRNA5-1	GATTTGGACACGGACCTCGCCGG	19G170100	20	0	0.0%	45	0	0.0%
			03G168700		0	0.0%		0	0.0%
	gRNA5-2	GCCGCCCCAAGTGTAAGCATCGG	19G170100	20	9	45.0%	30	6	20.0%
			03G168700		10	50.0%		10	33.3%
	gRNA5-3	GCCACACCGATGCTTACACTTGG	19G170100	20	1	5.0%	Not selected		
			03G168700		1	5.0%			
	gRNA5-4	GCTGGTGCATCCCGGGTTATTGG	19G170100	20	5	25.0%			
			03G168700		2	10.0%			
	gRNA5-5	GGTCTCTTCCCCTGTATTCTTGG	19G170100	20	3	15.0%			
			03G168700		5	25.0%			
Unpublished	gRNA6-1	GCTGGCCCTGTATTTACAAATGG	13G297700	20	1	5.0%	37	1	2.7%
			12G203900		2	10.0%		1	2.7%
	gRNA6-2	GTTTGGCCGCGGCCGTATAGGGG	13G297700	20	10	50.0%	42	8	19.1%
			12G203900		7	35.0%		9	21.4%
	gRNA6-3	GTATGACAACCCCTACTTGGTGG	13G297700	20	0	0.0%	Not selected		
			12G203900		0	0.0%			

**Table 5:** Gene-editing efficiency of gRNA7-14 in whole-plant transformation.

Code of Target Genes or Gene Pairs	Code of gRNAs	Gene Locus	Whole-Plant Transformation		
			# Cas9+ Plants	Mutant Events	Editing Rate (%)
7	gRNA7-1	18G003900	26	7	26.9%
		11G253000		4	15.4%
8	gRNA8-1	08G163900	11	2	18.2%
		15G263300		1	9.1%
9	gRNA9-1	10G009200	10	6	60.0%
		02G008600		8	80.0%
10	gRNA10-1	04G044000	27	8	29.6%
		06G044200		10	37.0%
11	gRNA11	04G003200	11	1	9.1%
12	gRNA12	05G012300	20	3	15.0%
13	gRNA13	13G161900	28	11	39.3%
14	gRNA14	20G012000	61	22	36.1%

## Data Availability

The data is contained within the manuscript and Supplementary Materials.

## Supplementary Materials

The following are available online at https://www.mdpi.com/article/10.3390/plants12051017/s1, Figure S1. Sequencing results of the targeted region in transgenic hairy roots; Figure S2. *GmPDS* sequencing results of the targeted region in transgenic hairy roots; Figure S3. Sequencing results of target regions of transgenic hairy roots for the selected sgRNA; Table S1. Correlation of editing efficiency of hairy root transformation and whole plant transformation; Table S2. Primers used in this study.
