# Supplementary material for: Combination of Hairy Root and Whole-Plant Transformation Protocols to Achieve Efficient CRISPR/Cas9 Genome Editing in Soybean
Source: Plants (Basel). 2023 Feb 23;12(5):1017. doi: 10.3390/plants12051017 (PMC10005656; doi:10.3390/plants12051017)
Supplement: Supplementary file 1 [file plants-12-01017-s001.zip › Supplementary Figures.pdf]

plants

Supplementary Materials

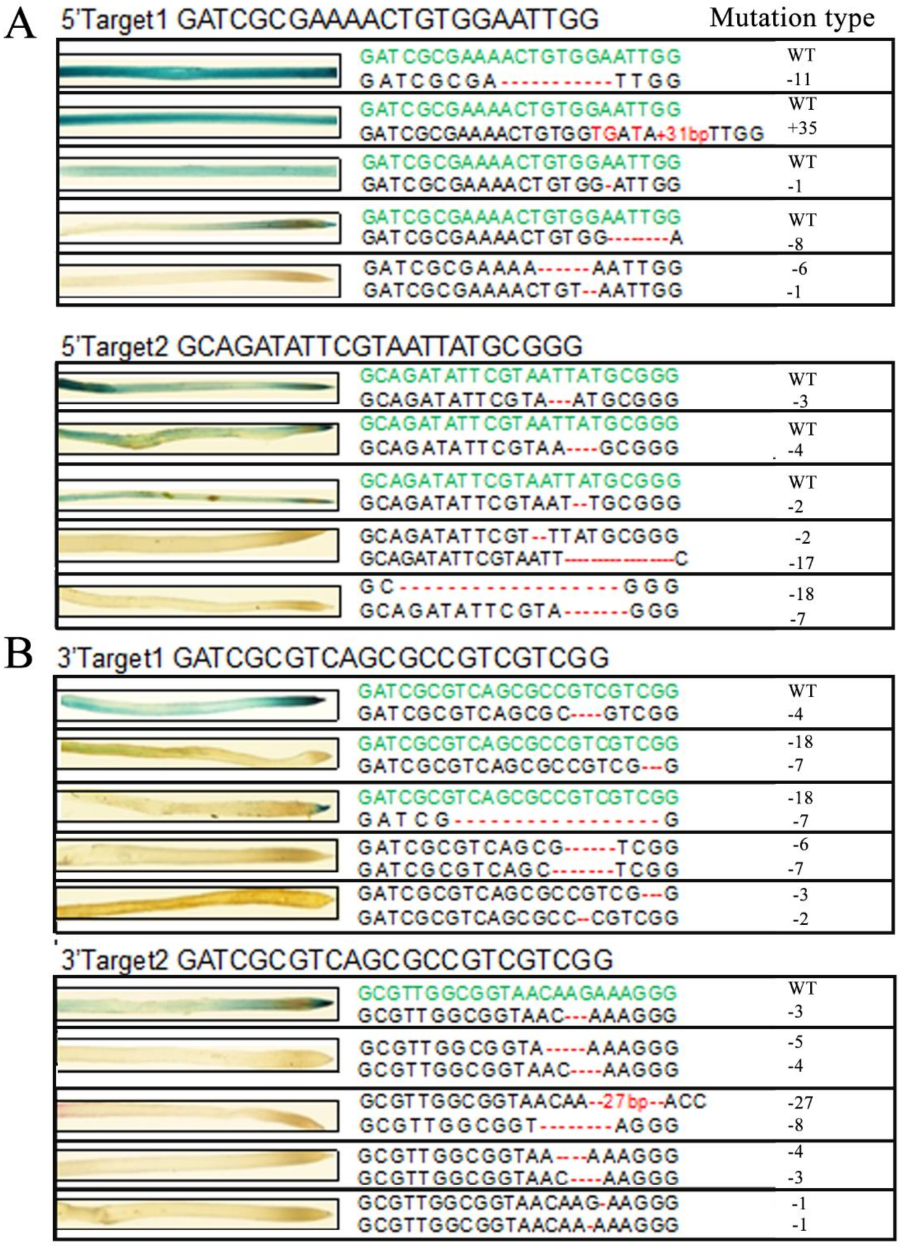

Figure S1. Sequencing results of the targeted region in transgenic hairy roots. (A) Target sequences were designed to the 5′ end of the *GUS* gene. (B) Target sequences were designed to the 3′ end of the *GUS* gene. Each hairy root is an independent event. Base substitutions are indicated with orange capital letters. The targeted sequences are shown on the top. The genotype of the mutation is indicated at the right of each sequence. -n, nucleotide deletion of the indicated number; +n, nucleotide of the inserted number; Percentages next to sequences indicate the number of amplicons with sequence over the number of total amplicons sequenced. There were 10 amplicons per event from the 5′-target and 3′-target events, respectively.

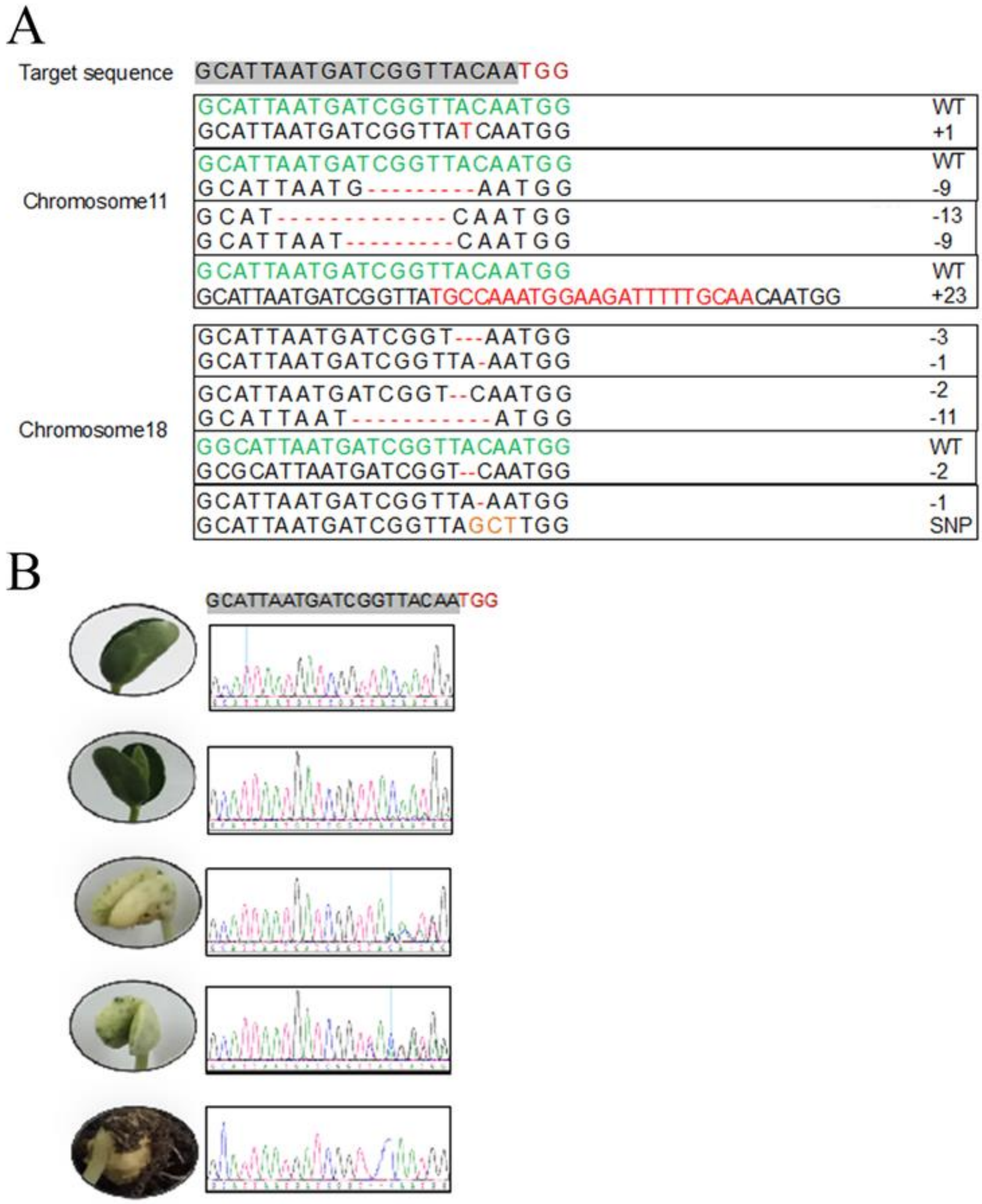

Figure S2. GmPDS sequencing results of the targeted region in transgenic hairy roots. (A) Sequencing of Cas9-induced GmPDS mutation in the T0 transgenic soybean plants. Wild-type sequences are in green, deletions and insertions are shown in red. Base substitutions are indicated with orange capital letters. The targeted sequences are shown on the top. -n, number of nucleotide deleted; +n, number of nucleotide inserted; Percentages next to the sequences is the ratio of amplicons with this sequence over the number of total amplicons sequenced. Ten amplicons per event were sequenced. (B) Sequencing analysis of GmPDS-CRISPR/Cas9 T1 soybean plants.

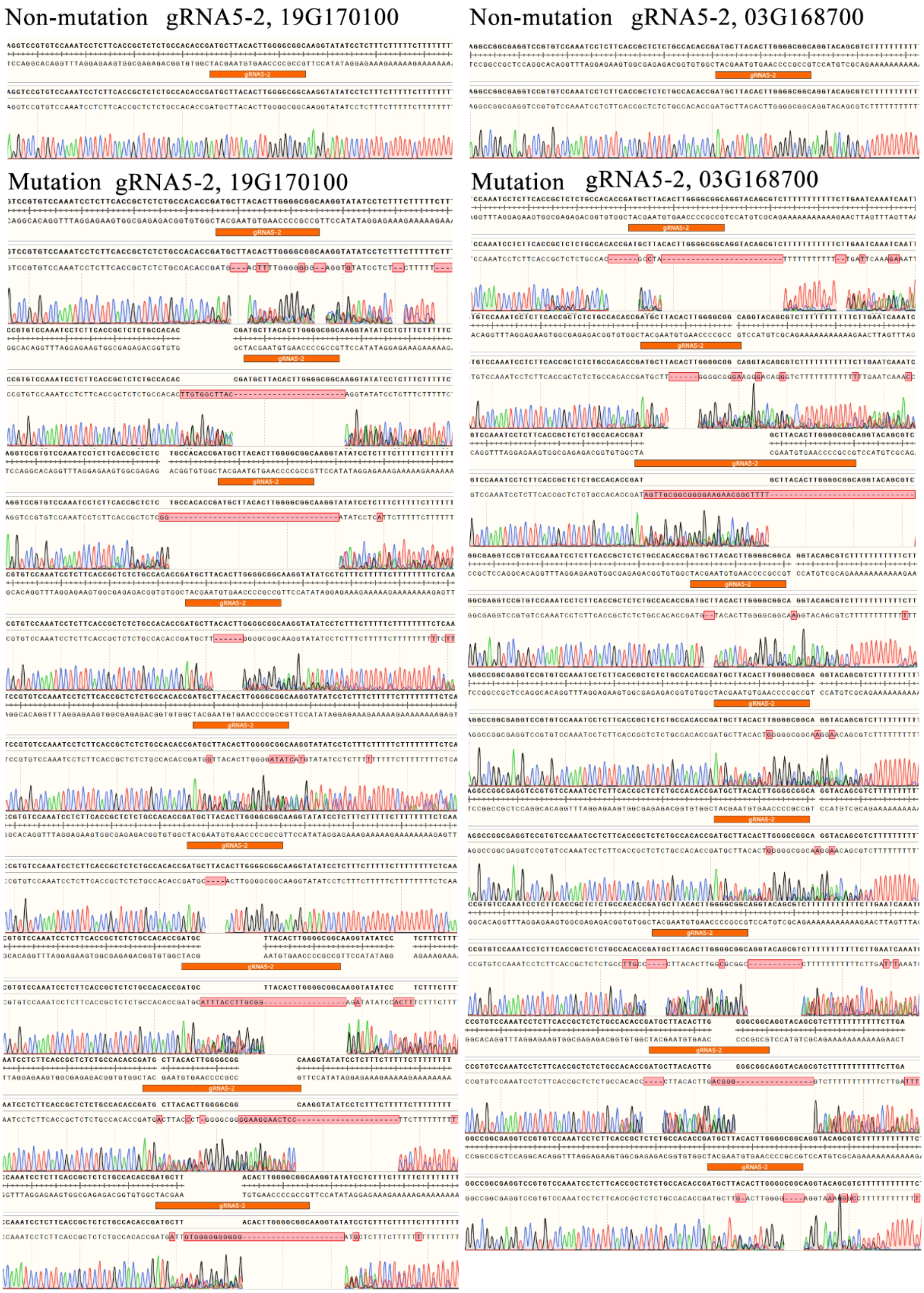
Non-mutation gRNA5-2, 19G170100
Non-mutation gRNA5-2, 03G168700
Mutation gRNA5-2, 19G170100
Mutation gRNA5-2, 03G168700

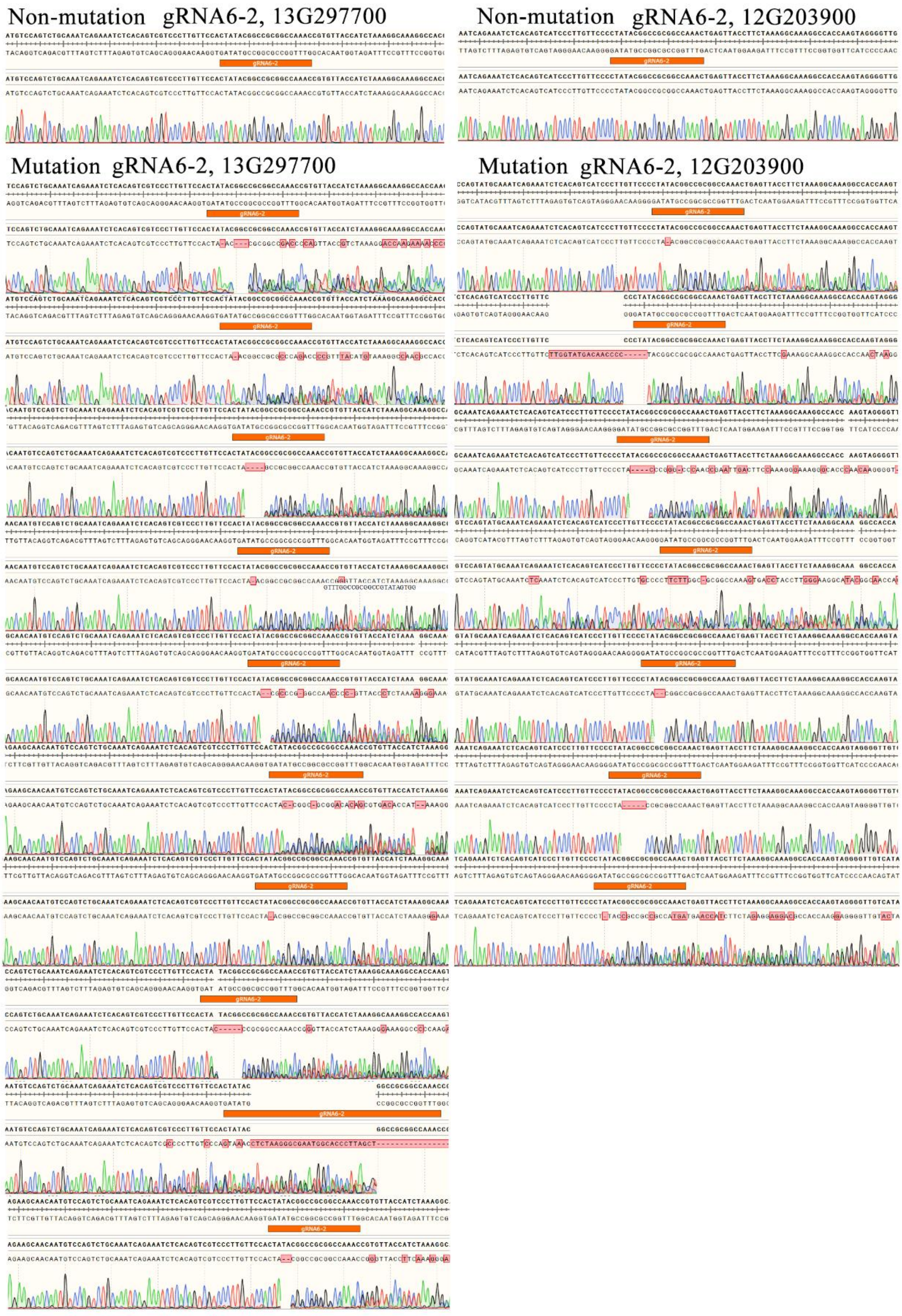

Figure S3. Sequencing results of target regions of transgenic hairy roots for the selected sgRNA. Results showed the sequences alignment of selected sgRNA5-2 (target of soybean *Glyma19G170100* and *Glyma03G168700)* and sgRNA6-2 (target of soybean *Glyma13G297700* and *Glyma12G203900)*. The target sequence locations were shown in the orange rectangle.
